# Risk factors associated with cystic ovarian disease in Norwegian dairy cattle

**DOI:** 10.1186/1751-0147-52-60

**Published:** 2010-11-08

**Authors:** Sindre T Nelson, Adam D Martin, Olav Østerås

**Affiliations:** 1 Department of Production Animal Clinical Sciences, Norwegian School of Veterinary Science, Oslo, Norway

## Abstract

**Background:**

The aims of this study were to establish the incidence of cystic ovarian disease (COD) and its geographical and seasonal variation in Norway, investigate the effect of COD on culling rates, and describe the effects of COD on subsequent reproductive performance and its association to twins.

**Methods:**

Diagnosis of COD was made by veterinary surgeons in the field. Four statistical models were made all including herd as random effect: The four different dependent variables investigated were: 1) Diagnosis of COD between 40 and 165 days in milk or not; (n = 511,657); 2) Twins or singleton; data restricted to lactations with new calving (n = 156,661): 3) Culling/removal or not (n = 573,184): 4) Culling due to reproductive problems; data included only lactations which ended in culling (n = 234,232). Model 1, 3 and 4 applied Cox regression models, and model 2 logistic regression. Independent variables were parity, twins/singletons, calving season, herd size, region, COD occurrence in present lactation (if not dependent), and COD diagnosis in previous lactation.

**Results:**

The incidence was 0.82% per lactation. COD increased with increasing parity, was smallest at herd size between 35 and 85 cows. Cows in 1^st ^parity and calved in spring had lowest hazard of COD and hazard for COD diagnosis was highest in autumn with HR = 2.6 (1.9 - 3.4) compared to spring. There was an interaction between parity and season. COD incidence was lower south of 60°N. Cows which experienced COD had an increased odds of giving birth to twins OR = 2.2 (1.7 - 2.7). Of those that were culled, those with COD were culled more frequently because of reproductive problems; HR = 2.1 (1.9 - 2.3) for higher parity than 2. Having COD diagnosed in the preceding lactation was a hazard for diagnosis in the lactation studied.

**Conclusion:**

COD diagnosis is strongly associated with season (autumn calving) and parity. Herds north of 60°N have more COD. Occurrence of COD is associated with twin births as well as culling due to reproduction.

## Background

Poor reproductive performance is one of the greatest causes of economic loss in dairy cattle production worldwide. Cystic ovarian disease (COD) is a cause of temporary infertility and one of the most common reproductive disorders in dairy cows with a reported incidence of 6 to 23% [1-6]. A meta-analysis published in 1999 showed that COD was associated with a 6 to 11 day increase in the calving to first service interval, and a 20 to 30 day increase in the calving to pregnancy interval [7]. It is also known to increase the risk of culling [8,9]. The economic impact of COD was calculated in 1986 to be $137 per case; but national, regional and seasonal variations were large [9]. A Swedish study found a case of COD decreased the net return between 470,- and 720,- SEK per cow per lactation [8]. It is economically beneficial to treat COD [10].

A number of cow level factors have been associated with an increased risk of COD. These include parity, constitutional weakness, body condition score, and genetic factors [4,5,11]. Milk yield has been identified as a risk factor in some studies but not in others. It is likely that this effect is dependent on the energy status of the cow, rather than the milk yield per se. High milk yield may contribute to negative energy balance which, when severe, results in metabolic and hormonal adaptations which influence follicle growth and cyst development [12]. Occurrence of COD predisposes an animal to COD in the following lactation [11]. Several studies have found that COD is associated with twinning [13-15].

Environmental factors including nutrition, feeding management, and housing type, are associated with risk of COD [12,16]. A seasonal effect on COD incidence has been identified. A study performed in North-East Spain (40°N) showed that cows calving in the summer are 2.6 times more likely to develop COD than cows calving in the winter, it attributed the difference to the seasonal variation of heat stress [11]. A Norwegian study found that both photo intensity and photoperiod were associated with reproductive performance [17]. Norway's latitude is between 58°N to 71°N and its climate much colder than that of North-East Spain. It is, therefore, reasonable to investigate geographical and seasonal variation of COD incidence in these different conditions.

Despite COD being a well known reproductive disorder, there is little new field data on its incidence and effects on reproduction, and there is still a discussion about which risk factors are predisposing [12]. The last population level study in the Nordic countries was performed over 20 years ago [18]. Therefore, the aims of this study were to: i) establish the incidence of COD for Norwegian Red cattle and its geographical and seasonal variation; ii) investigate the effect of COD on culling rates; and iii) describe the effects of COD on subsequent reproductive performance and association with twinning.

## Methods

### Study population

Data on the study population were extracted from all herds (approximately 15,000) which were members of the Norwegian Dairy Health Recording System (NDHRS) in the years 2005, 2006, 2007 and 2008. The Norwegian dairy population consists of 94% purebred Norwegian Red cattle. All other breeds were excluded from this study. All lactations starting with a calving during 2006 and 2007 were extracted including calving date (starting day), date of subsequent calving or date of removal from the herd. Lactation length was estimated as the distance from each calving until 15 days before the next calving or removal from the herd. If the lactation length was more than 516 days, that lactation was excluded. The number of included lactations was 579,722.

All recordings of COD were merged into the lactation in which they occurred. Each COD event, and day of diagnosis, was numbered from one onward according to the number of days in milk (DIM). Additionally, COD events in the previous lactation were merged into this dataset. In the same dataset, all service data were merged with the corresponding lactation and numbered according to DIM. Other variables included, and used in this study, were number of calves born, dystocia, date of birth, farm identity, date of culling, primary and secondary given reason for culling and breed.

### Case definition

The definition of COD in this paper is a diagnosis of COD that has been recorded on the animal's health card in the NDHRS made by practicing veterinary surgeons during their regular field work. This could have been a scheduled examination, in a reproductive herd health programme, or because the farmer wanted a specific cow examined. The NDHRS has been in place for more than 30 years and has been evaluated by Østerås et al. (2007) [19]. In this study COD was diagnosed according to guidelines from the Norwegian Cattle Health Services (NCHS) which defines COD as a "follicle-like structure identified by rectal palpation with a diameter of at least 2.5 cm in the absence of luteal tissue".

### Time to event calculations

Calving season was grouped into four classes; spring (March, April and May), summer (June, July and August), autumn (September, October and November) and winter (December, January and February). Each herd was categorised into one of 3 geographic areas according to the placement south-north (roughly according to latitude limit 60°N, 65°N and 70°N).

The distance from calving to COD diagnosis and between COD diagnoses within the same lactation were calculated. In addition, the time from calving to breeding, the time COD diagnoses to breeding, as well as between breeding intervals within the same lactation were calculated.

### Statistical analysis

Extracted data were analysed in SAS, version 9.1.3 (SAS Institute INC., Cary, NC). Calculation of descriptive statistics was performed using Proc Means or Proc Univariate. The distribution of data was evaluated using statistical and graphical methods. Possible correlations between variables were assessed by Chi-square for class variables, and simple regression for continuous variables.

Four multivariable models were made with presence or absence of COD diagnosis, twins at next calving, culling during lactation and reproduction given as reason for culling when culled, respectively as dependant variables. The models with COD, culling during lactation and reproduction given as reason for culling were constructed using Cox regression models applying Proc Phreg in SAS. The model with the dependant variable twins or not applied logistic regression using Proc Genmod.

In the model analysing COD as dependent variable, all lactations which ended with culling before 40 DIM, or those having COD diagnosed before 40 DIM, were excluded. The observation time ended at first event of COD, and lactations were censored at culling or at 165 DIM if not culled yet. Thus, a cow which first experienced COD after 165 DIM was classified as not having COD in this model. The time interval of 40 to 165 DIM was chosen as it covers the 10^th ^and 90^th ^percentiles from calving to first diagnosis for >2^nd ^parity and 1^st ^parity, respectively (Table 1). Lactations which began with the calving defined observation time in this analysis started at Day 40. The final model contained 3,913 events of COD within 511,657 lactations from 14,405 herds. The model was:

**Table 1:** The occurrence of cystic ovarian disease in days after calving stratified by parity

Parity	Time interval	n	Distribution in days				
			
			10%	25%	median	75%	90%
1^st^	Calving to 1^st ^diagnosis	845	49	70	93	124	164
2^nd^		1251	43	60	83	113	144
> 2^nd^		2662	40	56	76	106	139
1^st^	Calving to 2^nd ^diagnosis	79	72	99	123	157	214
2^nd^		93	78	87	109	142	204
> 2^nd^		279	62	78	100	128	158
1^st^	1^st ^to 2^nd ^diagnosis	79	5	11	18	33	63
2^nd^		93	10	11	18	33	63
> 2^nd^		279	10	13	21	32	58

Model 1: λ(*t*; Z_ki_) = λ_0_(*t*)e^*β'*^^Z^^*ki*^^(^^*t*^^) ^; where λ_0_(*t*) is an arbitrary baseline hazard function, β is the vector of regression coefficients; Z_*ki*_(*t*) is the covariate process associated with the k^*th *^lactation of the i^*th *^herd. The β is estimated by the maximum partial likelihood under the independent working assumption, and uses a robust sandwich covariance estimate to account for the intra-cluster dependence, as described by Lee et al. [20]. The tested independent variables included in the β vector were parity (1, 2, >2), herd size (<35, 35-84, >84 cows), calving season (four categories: spring = March, April and May; summer = June, July and August; autumn = September, October and November; winter = December, January and February), twins or singleton at birth (start of lactation), region in Norway was divided in three according to county borders fitting approximately at latitudes 60 and 65°N (south of 60°N; middle - between 60°N and 65°N; and north between 65°N and 70°N), as well as interactions between parity and calving season, and calving season and geographic region. Herd size was transformed into a class variable after identifying the parable shape of the association with a minimum between 35 to 85 cows in the herd. All class variables were transformed to separate class variables with parity 1, spring calving, herd size 35 to 85 cows, region South of 60°N as reference group. Including parity and season as interaction calving in spring at 1^st ^parity was made reference in the final model. COD in previous lactation and lactation starting with twins or singleton was also tested in the model. Independent variables or interaction terms with *P*-value > 0.05 were excluded from the model by the backward elimination procedure. The model fit was evaluated by plotting the deviance residuals against the covariates [21]. To test for the proportional hazard assumption, the log of the negative log of survival was plotted against time with most important independent variable as strata.

The model for estimating the probability of twins versus singleton was made from a sub-dataset including 156,661 lactations which ended in a new calving and this calving had a singleton or twins (n = 5,212). The model constructed was:

Model 2: logit(TWINS_i_) = β_0 _+ β_1_*parity_i_+ β_3_*(X_1i_) +....+ β_k_*(X_ki_) + μ_herd(i)_

The tested independent variables were the same as Model 1 in addition to twins, singleton or other remarks at birth (start of lactation) and COD diagnosis, or not, during the lactation. Variables with *P*-values > 0.05 were excluded from the model. Herd identity was introduced as random effect to adjust for correlated observation within each herd. This was done by implementing alternating logistic regression [22].

Model 3, the model for estimating the probability of removal versus remained in the herd (calved again), was estimated using a subset of the main dataset. This subset included 573,184 lactations that ended in calving or removal. Number of removals was 234,232 corresponding to 40.9%. These lactations were within 14,572 different herds. The model constructed was the same as the Cox regression model, Model 1. However, the dependant variable in Model 3 was time from calving until culling. Lactations not ending in culling were censored at the earliest of; the cow was sold as live animal, or the farmer ended the membership of NDHRS, or latest at 350 DIM. Intra-cluster dependence within herd was adjusted for using the procedure described for Model 1. Due to the non-proportional hazard in different parities, the model had to be run separately for parity 1, 2 and > 2, respectively. The tested independent variables were the same as for Model 1, including also COD diagnosis during lactation or not. Variables with *P*-values > 0.05 were excluded from the model. However, occurrence of COD was forced into the model.

The model for the probability of removal due to reproductive problems of those lactations ending in removal from the herd, versus the lactations ending in removal from reasons other than reproduction, was estimated using a subset of the main dataset. A total of 234,232 lactations were included, of which 35,827 (corresponding to 15.3%) was due to reproduction problems. These lactations were within 14,515 different herds. The model constructed was the same model as Model 1, however using time from calving until culling due to reproductive problems as dependent variable. Lactations not ending in culling due to reproductive problems were censored at the time the cow culled due to other reasons or at 350DIM, whatever came first. However, all cows were culled during the lactation of study. Intra-cluster dependence within herd was adjusted for using the procedure described for Model 1. Due to non-proportional hazard in different parities in culling hazard and different association to COD, the model had to be run separately for parity 1, 2 and > 2, respectively. The tested independent variables were the same as in Model 3. Variables with *P*-value > 0.05 were excluded from the model.

## Results

Data were available from 14,572 herds with mean herd size of 17.9 cow years. The general production level was 6,388 kg milk pr cow year in 2006 and 6,543 kg milk per cow year in 2007. The parity distribution was; 1^st ^parity: 36.9%, 2^nd^: 26.7%, 3^rd^: 17.2%, 4^th^: 9.9%, > 4^th^: 9.3%. The mean lactation length was 303 days (SD 121 days), whilst the median lactation length was 347 days.

In total, 4758 lactations had one or more COD registrations (incidence 0.82%). The percentage of lactations with COD diagnosis prior to birth of a singleton calf was 0.83, and prior to the birth of twin calves 1.08. None of the 45 lactations that began with the birth of triplets had COD diagnosed in the previous lactation. The majority of COD containing lactations had a single COD occurrence (89.3%, n = 4249). However, 451 animals (9.5%) had a 2^nd^, 49 animals (1.0%) a 3^rd^, and 9 (0.2%) animals had a 4^th ^COD registration within the same lactation. For cows with no records of COD during lactation the mean calving to first service interval was 81 days, with 90% occurring between 41 and 142 days. For cows which experienced COD during the lactation the mean calving to first service interval was 87 days, with 90% occurring between 42 and 150 days.

In total 55.6% of the cows that were diagnosed with COD began a new lactation, compared to 59.2% that did not have COD. Of the cows that calved again, and did not have COD, had a calving to last service interval of 96 days (with 90% between 47 and 173 days). Those with COD had calving to last service interval of 116 days (with 90% between 61 and 193 days). For cows which did not calve again (removed or culled) the corresponding figures were 114 (48 and 219) days, and 131 (59 and 238) days, respectively.

The distribution of parity, calving season, geographic distribution, number of offspring, lactations ending in calving or removal, and the recorded number of COD for each category are presented in Table 2. The incidence of COD was associated with herd size. Herds with the lowest incidence of COD were those with 35-85 cows per annum. The regions had similar patterns of COD occurrence throughout the year. The highest incidence was in autumn calving cows.

**Table 2:** Distribution of lactations with cystic ovarian disease by parity, calving season, geographic distribution and culling

Variable	Class	N lactations	n	COD %
Total material		597,722	4,758	0.82

Parity	1	213,006	845	0.40
	2	153,532	1251	0.81
	3	99,647	1211	1.21
	4	56,706	780	1.36
	> 4	53,073	671	1.25

Calving season	December, January, February	115,394	834	0.72
	March, April, and May	135,188	525	0.39
	June, July and August	141,096	1,025	0.72
	September, October, and November	183,356	2,374	1.28

Geographic distribution	Østfold, Akershus, Vestfold	27,751	132	0.47
	Hedmark, Oppland, and Buskerud	118,559	1,109	0.93
	Telemark, and Agder	22,330	130	0.58
	Rogaland	99,651	635	0.63
	Hordaland, Sogn og Fjordane	68,616	649	0.94
	Møre og Romsdal and Trøndelag	181,556	1,635	0.89
	Nord-Norge	56,451	468	0.82

Lactation ended with:	New calving	340,498	2,643	0.77
	Culling	234,466	2,115	0.89

The distances from calving to first and second COD recording, as well as the distances between first and second COD in parities 1, 2 and above 2 are presented in Table 1. The proportion of lactations with COD stratified by culling and if they had COD diagnosed in the previous lactation, is presented in Table 3. Of those cows that were culled, the farmers gave the primary reason for culling to be reproductive problems for 827 cows (39.1%) for those that had COD, and 32,378 (13.8%) for those without COD. Where given the corresponding figures for the secondary reason of culling were 89 (4.2%) and 2668 (1.1%), respectively.

**Table 3:** Lactations with cystic ovarian disease by diagnosis in previous lactation, stratified by culling

Calved again	COD or not in previous lactation	COD in next lactation	N*	Percentage
Yes	Yes	Yes	78	12. 6
		No	543	87.4
	No	Yes	2,043	1.0
		No	200,849	99.0

Sum		Yes	2,121	1.0
		No	201,392	99.0

No	Yes	Yes	60	9.3
		No	588	90.7
	No	Yes	1,732	1.1
		No	159,978	98.9

Sum		Yes	1,792	1.1
		No	160,566	98.9

* First parity excluded

The multivariable Cox survival model confirmed associations between COD occurrence and COD in previous lactation, parity, herd size, calving season as well as interactions between parity and calving season, and geographical region (Table 4). There was no association to twin or singleton at birth before COD diagnosis. The interaction between calving season and geographical region approached significance (*P *= 0.07), and so removed from the final model. The interaction between parity and season was highly significant with much higher COD risk in higher parities for cows calved during the autumn.

**Table 4:** Estimated hazard ratio from the multivariable Cox regression model with time of cystic ovarian disease (COD) diagnosis between day 40 and 146 in lactation and herd as cluster

Variable	Class	N	Chi-square	HR (95% CI)
COD recorded during previous lactation	No	510,565	-	1.0
	Yes	1,092	364.3	7.5 (6.1 - 9.3)
Interaction parity and calving season	1^st ^and spring	35,969	-	1,0
	1^st ^and summer	45,126	24.7	2.1 (1.6 - 2.8)
	1^st ^and autumn	64,667	44.8	2.6 (1.9 - 3.4)
	1^st ^and winter	40.776	11.0	1.7 (1.2 - 2.3)
	2^nd ^and spring	32.530	7.3	1.6 (1.1 - 2.2)
	2^nd ^and summer	31,007	72.4	3.5 (2.6 - 4.7)
	2^nd ^and autumn	43,755	168.8	6.1 (4.6 - 7.9)
	2^nd ^and winter	29,121	85.7	3.9 (2.9 - 5.2)
	> 2^nd ^and spring	50,802	43.5	2.6 (2.0 - 3.4)
	> 2^nd ^and summer	45,134	148.4	5.5 (4.2 - 7.3)
	> 2^nd ^and autumn	56,931	300,0	10.2 (7.8 - 13.2)
	> 2^nd ^and winter	32,840	146.1	5.5 (4.1 - 7.2)
Herd size	< 35	430,962	15.8	1.4 (1.2 - 1.6)
	35 to 84	75,430	-	1.0
	> 84	5,265	7.7	2.0 (1.2 - 3.3)
Region	< 60° latitude	131,159	-	1.0
	60 to 65 °	329,186	21.6	1.3 (1.2 - 1.5)
	> 65 °	51,312	6.1	1.2 (1.0 - 1.4)

Results from the multivariable logistic regression model describing the risks of giving birth to twins were: twins or singleton at the start of lactation, parity, COD occurrence, region and calving season, in this ranking order according to Chi-square values. Herd had a small cluster effect. The detailed results are presented in Table 5. The lactations with COD had an OR = 2.2 (1.7 - 2.7) of having twins at the next calving compared to lactations without COD.

**Table 5:** Estimates from the multivariable logistic model of having twins versus singleton as dependent variable

Variable	Class	N	Estimate (stderr)	OR (95% CI)
Intercept		156,661	-3.39 (0.04)	
Parity	1	63,038	0.00	1.0
	2	44,182	0.20 (0.04)	1.2 (1.1 - 1.3)
	> 2	49,441	0.32 (0.03)	1.4 (1.3 - 1.5)
Diagnosis of COD during lactation	No	155,455	0.00	1.0
	Yes	1,206	0.77 (0.11)	2.2 (1.7 - 2.7)
Lactation start with	Singleton	147,096	0.00	1.0
	Twins	2902	1.09 (0.07)	3.0 (2.6 - 3.4)
	Triplets	7	1.46 (1.12)	4.3 (0.5 - 39)
	Others*	6,656	0.05 (0.07)	1.1 (0.9 - 1.2)
Calving season	March-May (spring)	37,141	0.00	1.0
	September-November (autumn)	49,171	- 0.14 (0.04)	0.9 (0.8 - 0.9)
	December-February (winter)	30,545	- 0.08 (0.04)	0.9 (0.8 - 1.0)
	June-August (summer)	39,804	0.06 (0.04)	1.1 (1.0 - 1.2)
Region	< 60° latitude	39,175	0.00	1.0
	60 to 65 °	102,066	- 0.20 (0.03)	0.8 (0.8 - 0.9)
	> 65 °	15,420	- 0.27 (0.06)	0.8 (0.7 - 0.9)
Herd effect		13,080	0.06 (0.03)	1.1 (1.0 - 1.1)

*Lactations starting with an abortion or lactations starting with calving prior to the date the cow was introduced into the present herd

Results from the multivariable Cox survival model describing the hazards for removal from the herd were (in order of importance, *P *< 0.001 for all variables): parity, singleton or twin at lactation start, calving season, herd size, region and finally COD in previous lactation, but not so in present lactation. Detailed estimates are presented in Table 6. The lactations with COD the previous lactation had a HR = 1.2 (1.0 - 1.5) of ending in removal in 2^nd ^lactation compared to lactations without COD.

**Table 6:** Estimated hazard ratio from the multivariable Cox regression model with time at culling as the dependent variable and herd as cluster. COD = cystic ovarian disease

		**1**^**st **^**parity**			**2**^**nd **^**parity**			**>2**^**nd **^**parity**		
**Variable**	**Class**	**N**	**Chi-square**	**HR (95% CI)**	**N**	**Chi-square**	**HR (95% CI)**	**N**	**Chi-square**	**HR (95% CI)**

COD recorded during previous lactation	No	197,900	-	-	144,464	-	1.0	199,409	-	1.0
	Yes	0	-	-	256	3.7	1.22 (1.00-1.49)	952	18.7	1.22 (1.12-1.34)
COD recorded during present lactation	No	197,105	-	1.0	143,517	-	1.0	197,792	-	1.0
	Yes	795	1.8	0.91 (0.80-1.04)	1,203	2.3	1.07 (0.98-1.17)	2,569	59.2	0.79 (0.75-0.84)
Twins or triplets	No	196,303	-	1.0	140,825	-	1.0	192,986	-	1.0
	Yes	1,597	5.2	1.11 (1.02-1.22)	3,895	67.6	1.25 (1.19-1.32)	7,375	162.2	1.25 (1.20-1.29)
Calving season	Spring	38,384	103.7	1.17 (1.14-1.21)	33,772	2.9	1.03 (1.00-1.06)	54,731	4.3	1.02 (1.00-1.05)
	Summer	47,907	-	1.0	35,491	-	1.0	48,959	-	1.0
	Autumn	68,261	20.5	1.07 (1.04-1.09)	45,229	23.5	0.93 (0.91-0.96)	61,351	71.6	0.92 (0.90-0.93)
	Winter	43,348	183.9	1.22 (1.19-1.26)	30,228	25.2	1.08 (1.05-1.11)	35,320	35.7	1.07 (1.05-1.10)
Herd size	< 35	165,420	-	1.0	121,395	-	n.s	169,781	-	n.s
	35 to 84	30,489	7.9	1.06 (1.02-1.10)	21,834	-	n.s.	28,555	-	n.s.
	> 84	2,047	0.4	1.06 (0.88-1.27)	1,536	-	n.s.	2,088	-	n.s.
Region	< 60° latitude	53,392	34.0	1.15 (1.10-1.20)	37,822	22.3	1.11 (1.06-1.16)	49,708	10.8	1.06 (1.02-1.09)
	60 to 65 °	125,037	8.1	1.06 (1.02-1.11)	92,371	1.4	1.02 (0.99-1.06)	130,883	0.4	1.01 (0.98-1.04)
	> 65 °	19,471	-	1.0	14,527	-	1.0	19,770	-	1.0

Results from the multivariable model describing the hazards for removal from the herd due to reproductive problems revealed the following hazards (in order of importance): COD both in the previous and the present lactation, calving season, region, twins or singleton at birth and herd size. Detailed estimates are presented in Table 7. Of the lactations ending in removal, those with COD had a HR = 1.4 to 2.1 of ending lactation due to reproduction problems compared with ending the lactation due to other reasons in the 1^st ^to > 2^nd ^parity, respectively.

**Table 7:** Estimated hazard ratio from the multivariable Cox regression model with time at culling due to reproductive problems amongst only culled cows and herd as cluster

		**1**^**st **^**parity**			**2**^**nd **^**parity**			**>2**^**nd **^**parity**		
**Variable**	**Class**	**N**	**Chi-square**	**HR (95% CI)**	**N**	**Chi-square**	**HR (95% CI)**	**N**	**Chi-square**	**HR (95% CI)**

COD recorded during previous lactation	No	50,375			42,601	n.s		81,680	-	1.0
	Yes	0			90	n.s		442	14.2	1.56 (1.24-1.97)
COD recorded during present lactation	No	50,173	-	1.0	42,288	-	1.0	81,169	-	1.0
	Yes	202	11.9	1.38 (1.15-1.66)	403	45.3	1.68 (1.45-1.96)	953	178.3	2.06 (1.85-2.29)
Twins or triplets	No	49,927	n.s		41,302	-	1.0	78,539	n.s	
	Yes	448	n.s		1,389	4.8	1.15 (1.01-1.30)	3,583	n.s	
Calving season	Spring	10,328	-	1.0	10,229	-	1.0	23,002	-	1.0
	Summer	11,027	31.9	1.24 (1.15-1.35)	10,275	19.4	1.18 (1.10-1.27)	19,862	46.1	1.27 (1.18-1.36)
	Autumn	16,890	1.1	1.04 (0.97-1.11)	12,597	1.8	1.05 (0.98-1.12)	23,706	8.0	1.10 (1.03-1.17)
	Winter	12,130	2.8	1.06 (0.99-1.14)	9,590	0.5	1.02 (0.95-1.10)	15,552	2.7	1.06 (0.99-1.13)
Region	< 60° latitude	14,113	0.5	1.04 (0.94-1.15)	11,720	0.2	0.98 (0.88-1.09)	21,059	10.5	1.19 (1.07-1.33)
	60 to 65 °	31,567	7.7	1.14 (1.04-1.25)	26,789	6.7	1.14 (1.03-1.26)	52,941	18.6	1.24 (1.12-1.36)
	> 65 °	4,695	-	1.0	4,182	-	1.0	8,122	-	1.0

COD = cystic ovarian disease.

## Discussion

The incidence of COD in Norwegian Red cattle was found to be lower than the figure previously presented by Østerås et al. [19]. This could be, in part, due to a real decrease from 2005 to 2008, but it could also be due to calculation differences. This study reports incidence per lactation, whilst the earlier study reports incidence per 100 cow years.

### Definition

Older review papers define, COD as "anovulatory, follicular structures more than or equal to 2.5 cm in diameter that persists for more than 10 days", some include additional criteria such as "in absence of luteal tissue"  [2,3,23]. However, during the last decade the following definition has gained popularity: Ovarian follicle cysts at least 17 mm in diameter and persist for more than 6 days with no corpus luteum detectable by ultrasound [1]. The term COD should be reconsidered as the cystic follicles generally occur without obvious clinical signs [12]. The current study is based on the field work of practicing veterinary surgeons. Under these conditions repeat examination of the ovaries to see if the COD lasted for more than 10 days was not practical; therefore the NCHS definition was used.

The diagnoses in this study were mainly made by manual rectal ovarian palpation and they may have been the result of a scheduled examination, in a reproductive herd health programme, or because the farmer wanted a specific cow examined. Either way the identification of COD is strongly dependent on individual farmer's use of veterinary services. It is, therefore, important to include the random herd effect in the models. Consequentially, it is likely this study has a substantial degree of underreporting, because farmers do not present all cows with COD for veterinary examination. The study may have misclassified lactations in which COD was not detected. Currently the use of regular fertility examinations is sporadic in Norway and, as such, this paper probably reflects the extent to which COD is causing reproductive problems in the Norwegian dairy production. It is important to remember that normal follicular activity may have been incorrectly diagnosed as COD. Early developing corpora lutea, and some corpora lutea of pregnancy, are soft and can be misdiagnosed as cysts by manual rectal palpations [5].

The authors consider that the lack of diagnoses occurred more frequently than wrong diagnosis of COD in this study. Therefore the 'true' incidence of COD in the Norwegian Red is likely to be higher than reported in this paper, particularly as spontaneous recovery from COD occurs [11,23]. Furthermore, we consider that the diagnoses used in the current study will have a low sensitivity and a much higher specificity. The positive value of both follicular and luteal cysts diagnosed by manual rectal palpation has been reported to be 66%, compared to 75% and 85%, respectively, when diagnosis is made with the aid of transrectal ultrasonography [24]. If exposure variables were associated with herd, this could lead to different misclassification in different exposure groups. Such misclassification could lead to wrong interpretation of the results. However, in this study we consider the exposure variables parity, herd size, season and latitude to be independent of farmer. Therefore the misclassification bias should not be large. However, each misclassification will lead to a loss of statistical power in the analyses, and may result in a failure to identify associations. The authors consider the chance of missing associations in this study greater than the chance of identifying wrong associations. The size of the material will improve the study's power, assuming a lack of selection bias.

This study reflects the understanding of COD as experienced in the field, and shows the real effect of the diagnosis and treatment veterinary surgeons are performing. A further strength of this study is that the cows are examined in their every day environment and not under constructed or artificial conditions which may cause the animals stress, a possible risk factor for the disease [25-29].

### Statistical model

The cow population is very dynamic, demonstrated in this study by a herd removal rate of 40.8%. Given this fact, survival analysis was chosen as statistical method to be used for model with hazard having COD, culling and culling due to reproduction in the analyses, as it accounts for censored data. However, as cows within the same herd are not independent of each other, the marginal Cox model for clustered data was applied. For the hazard estimate of twins associated with COD alternative logistic regression was used [22] with herd included as a random effect to account for the dependency between observations within the same herd. To reduce possible selection bias the lactation period of estimating associations to COD was restricted to 40 to 165 DIM. For the culling models we tested if the results were different when the start of observation was changed from day of calving to 40 DIM or 150 DIM. All these test models were consistent with the published models. Therefore, we consider that the chosen starting point affect the results minimally

### Risk Factors

#### Parity

The likelihood of COD occurrence increases with parity. This may be the result of other pathological and physiological conditions which are related to increasing parity, e.g. milk fever [30]. Another factor may be selection bias, a farmer choosing to keep high yielding cows as long as possible in the herd [3,31]. Some studies have shown a correlation between high milk yield and COD occurrence [3,6] but other studies disagree [9,32,33]. Interestingly the occurrence of COD in animals in their third lactation or more is preventive for culling despite the reduced reproductive performance in these animals. This means that these animals must have other traits which the farmer values over and above the reduction in reproductive performance, e.g. high milk yield.

#### COD in the preceding lactation

Cows which experienced COD in their last lactation have a considerably higher hazard of suffering COD in the present lactation. This is understandable as there is evidence that COD is associated with the cow's genetics and environment [12]. However, when a decision is been taken as to whether to cull a cow that has COD or not, and when calculating the costs of the disease, the increased hazard of reoccurrence should be included.

#### Herd size

This study has demonstrated a correlation between herd size and COD occurrence. Cattle are gregarious animals with a diurnal rhythm and housing them can limit their natural behaviour. The exact effect of sub-optimal housing is not known, but a negative correlation with reproduction, including COD occurrence, is presumed. Simensen, et al. [16] found more COD diagnoses in free-stalls compared to tie-stalls [16]. Free-stalls are more frequent in larger herds. Hence, the identified herd size effect in the study could be an effect of housing system. Unfortunately, the information of housing system was not available in this study. A Belgian study asserts the housing of cows is a significant risk. Their study showed a 5.7 greater risk of getting COD when calving indoors compared to pasture calving [34].

#### Season

This study has shown that autumn calving increases COD occurrence, compared to calving in winter, spring and summer, which confirms the pattern found in Sweden in 1991 [18]. Cows would have their active reproduction period 40 to 170 days after calving according to this study. Thus, cows that calved during autumn will have their service period during the housed winter time. In tropical or hot regions there are reports of an opposite trend with more COD during summer [35,36], but studies from the USA did not find seasonality to be important [9,37]. However, to avoid or minimize stress for gregarious diurnal animals, light is important for daily tasks and for recognising herd mates. There is a higher culling rate due to reproductive problems for cows that calved during the autumn.

#### Latitude

Norway is a climatically and environmentally diverse country in which regional differences in COD occurrence could be expected. There was less COD south of 60°N compared to north of 60°N. North of 65°N there is constant daylight during the summer and no daylight during the winter. Despite farmers having artificial lighting, effects on reproductive performance do exist [17]. There could also be other differences between southern and northern Norway which explain this association, such as feed quality, feeding strategy, length of pasture time, etc. Moreover, it is interesting to observe that there is a seasonal as well as a latitude effect. Heat stress as observed in Spain[11] could also occur in Norway during summer, but not to the same extent as in Spain or other southern European countries.

#### Twins

The correlation between COD occurrence and singleton vs. twin calving supports the earlier study of Emmanuelson and Bendixen [18] which concluded that there is an increased risk of twinning after a COD occurrence. Silvia et al. have found that 47% of cows had two or more persistent follicles at the time of first detection [1,14], which also might explain the correlation between COD and twinning. Kinsel et al. [14] also found that increasing milk yield over time is the most important risk factor for twinning [14], and as described earlier, there is a correlation between milk yield and COD occurrence. Furthermore, in the present study, the association of twins is opposite directed with both season and latitude, compared to the association with COD. This point at different mechanisms, as there are less twins with cows calved during autumn and cows north of 60°N, while there are more COD. There is also a small herd effect for twins.

#### Removal/culling

The reason for the greater relative percentage of cows with COD in the cows being culled, compared to those staying in the herd may be because the COD occurrence is a considerable part of the reproductive problem. This was also confirmed in this study, using the given descriptive reason by the farmer in the analyses and concurs with the findings of Refsdal [38]. Norwegian herds also have a relatively high replacement percentage, and cows showing any abnormalities, e.g. COD occurrence [39], or poor milk yield are preselected as culling candidates, as these cows may have problems with getting pregnant at economic feasible time in lactation.

## Conclusion

Cows in higher parity have an increased risk of being diagnosed with COD. Cows treated for COD in one lactation have an increased risk of having a COD diagnosed in the following lactation. This study also illustrates that autumn calving in Norway is associated with higher risk of COD, especially for cows in higher parities. Cows with COD diagnosis had higher risk of giving birth to twins. Cows north of 60°N had higher risk of COD, and a lower risk of giving birth to twins. Season is associated in the opposite direction with twinning compared to the association COD. Cows treated for COD in the previous lactation had higher hazard for culling and those who were culled had a higher hazard of culling due to reproductive problems.

## Competing interests

The authors declare that they have no competing interests.

## Authors' contributions

STN, ADM and OØ have performed this work as a part of STN's European College of Bovine Health Management residency programme. All authors were involved in the study design, interpretation of results and drawing of conclusions. OØ performed the main statistical analyses, and STN was the head writer of the manuscript. All authors have been active in writing this paper and read and approved the final manuscript.
